# Post-transcriptional regulation of *SHANK3* expression by microRNAs related to multiple neuropsychiatric disorders

**DOI:** 10.1186/s13041-015-0165-3

**Published:** 2015-11-16

**Authors:** Su-Yeon Choi, Kaifang Pang, Joo Yeon Kim, Jae Ryun Ryu, Hyojin Kang, Zhandong Liu, Won-Ki Kim, Woong Sun, Hyun Kim, Kihoon Han

**Affiliations:** Department of Biological Sciences, Korea Advanced Institute of Science and Technology, Daejeon, 305-701 South Korea; Center for Synaptic Brain Dysfunctions, Institute for Basic Science, Daejeon, 305-701 South Korea; Jan and Dan Duncan Neurological Research Institute at Texas Children’s Hospital, Houston, 77030 USA; Department of Pediatrics, Baylor College of Medicine, Computational and Integrative Biomedical Research Center, Houston, 77030 USA; Department of Anatomy and Division of Brain Korea 21 Biomedical Science, College of Medicine, Korea University, Seoul, 136-705 South Korea; HPC-enabled Convergence Technology Research Division, Korea Institute of Science and Technology Information, Daejeon, 305-701 South Korea; Department of Neuroscience and Division of Brain Korea 21 Biomedical Science, College of Medicine, Korea University, Seoul, 136-705 South Korea

**Keywords:** *SHANK3*, Post-transcriptional regulation, microRNA, Dendritic spine, Bipolar disorder

## Abstract

**Background:**

Proper neuronal function requires tight control of gene dosage, and failure of this process underlies the pathogenesis of multiple neuropsychiatric disorders. The *SHANK3* gene encoding core scaffolding proteins at glutamatergic postsynapse is a typical dosage-sensitive gene, both deletions and duplications of which are associated with Phelan-McDermid syndrome, autism spectrum disorders, bipolar disorder, intellectual disability, or schizophrenia. However, the regulatory mechanism of *SHANK3* expression in neurons itself is poorly understood.

**Results:**

Here we show post-transcriptional regulation of *SHANK3* expression by three microRNAs (miRNAs), miR-7, miR-34a, and miR-504. Notably, the expression profiles of these miRNAs were previously shown to be altered in some neuropsychiatric disorders which are also associated with *SHANK3* dosage changes. These miRNAs regulated the expression of *SHANK3* and other genes encoding actin-related proteins that interact with Shank3, through direct binding sites in the 3′ untranslated region (UTR). Moreover, overexpression or inhibition of miR-7 and miR-504 affected the dendritic spines of the cultured hippocampal neurons in a Shank3-dependent manner. We further characterized miR-504 as it showed the most significant effect on both *SHANK3* expression and dendritic spines among the three miRNAs. Lentivirus-mediated overexpression of miR-504, which mimics its reported expression change in postmortem brain tissues of bipolar disorder, decreased endogenous Shank3 protein in cultured hippocampal neurons. We also revealed that miR-504 is expressed in the cortical and hippocampal regions of human and mouse brains.

**Conclusions:**

Our study provides new insight into the miRNA-mediated regulation of *SHANK3* expression, and its potential implication in multiple neuropsychiatric disorders associated with altered *SHANK3* and miRNA expression profiles.

**Electronic supplementary material:**

The online version of this article (doi:10.1186/s13041-015-0165-3) contains supplementary material, which is available to authorized users.

## Background

Accumulating evidence from both human and animal model studies suggests that genetic variants are critical risk factors for various neuropsychiatric disorders [1]. Copy number variations (CNVs), deletions and duplications, are structural variations in the genome that cause gene dosage changes. Interestingly, there are some loci in the human genome, such as 15q11-13, 16p11.2, and 22q13.3, both deletions and duplications in which are associated with neuropsychiatric disorders [2–4]. This suggests that the correct dosage of some genes within these loci is critical for normal brain function, and that there should be some regulatory mechanism in the brain to tightly control the expression of these dosage-sensitive genes.

The *SHANK3* (also called *ProSAP2*) gene, which encodes for core scaffolding proteins at the postsynaptic density (PSD) of glutamatergic synapses [5], is a typical dosage-sensitive gene in chromosome 22q13.3. Deletions and point mutations of *SHANK3* are associated with Phelan-McDermid syndrome, autism spectrum disorders (ASDs), intellectual disability, schizophrenia and bipolar disorder [6–8]. Moreover, its duplications are linked to Asperger syndrome, attention deficit hyperactivity disorder, schizophrenia and bipolar disorder [9–12]. Recent cell culture and animal model studies have revealed the molecular and cellular pathophysiology of the neuropsychiatric disorders caused by altered *SHANK3* dosage [12–19]. In contrast, however, the regulatory mechanism that underpins the tight control of *SHANK3* expression in neurons itself remains largely unknown.

MicroRNAs (miRNAs) are small non-coding RNAs that bind to the 3′ untranslated regions (3′UTRs) of target mRNAs and downregulate mRNA expression by reducing mRNA stability or by inhibiting translation [20]. As critical post-transcriptional regulators of gene expression, miRNAs are involved in widespread biological processes of the nervous system, in both physiological and pathological conditions, including neuronal development, synapse formation and plasticity, and neurodegeneration [21–24]. Furthermore, recent studies revealed altered miRNA expression profiles in postmortem brains or blood samples of patients with various neuropsychiatric disorders, including ASDs, schizophrenia, bipolar disorder and major depression [25–28]. In many cases, however, the causative roles of altered miRNA expression in neuropsychiatric disorders are not clear, because the key target genes and neuronal mechanisms affected by the miRNAs have not been identified. We reasoned that if there are miRNA target genes mediating pathogenesis, dosage-sensitive genes involved in neuronal function could be the most reasonable targets. Therefore, by investigating the relationship between the miRNAs and dosage-sensitive genes associated with the same type of neuropsychiatric disorder, we might gain some insight, not just into the pathogenesis of the disorder, but also into the miRNA-mediated regulation of dosage-sensitive genes.

In this study, we examine this possibility for the *SHANK3* gene and report post-transcriptional regulation of *SHANK3* expression by three miRNAs, miR-7, miR-34a, and miR-504, which were previously shown to be altered in some neuropsychiatric disorders that could also be caused by *SHANK3* dosage changes. We also show that these miRNAs regulate neuronal dendritic spines in a Shank3-dependent manner, which might provide some insight into the pathogenic mechanisms of neuropsychiatric disorders with altered miRNA expression profiles.

## Results

### miR-7, miR-34a, and miR-504 directly regulate the expression of *SHANK3*

Using the TargetScan prediction tool (Release 6.2, http://www.targetscan.org/vert_61/), we identified ~30 putative miRNA families that have evolutionarily conserved binding sites in the human *SHANK3* 3′UTR (Additional file 1: Table S1). Following a literature search, we narrowed down this list of miRNAs based on their neuronal expression, and their expression changes in the neuropsychiatric disorders which are also associated with *SHANK3* dosage changes. Finally, we chose three miRNAs, miR-7, miR-34a, and miR-504 because of their strong 8-mer type binding sites [20] in the *SHANK3* 3′UTR. The expression of miR-7, miR-34a and miR-504 were reported to be altered in the postmortem brains, fibroblasts, or blood samples of patients with schizophrenia, depression, or bipolar disorder (Additional file 1: Table S2) [29–34]. Recently, Zhang et al. claimed that the miR-7/*SHANK3* axis could be involved in schizophrenia pathogenesis, showing an inverse correlation between the expression levels of miR-7 and *SHANK3* [35]. However, neither the direct binding of miR-7 to the *SHANK3* 3′UTR nor its functional effect on neuronal synapses have been reported.

**Fig. 1:**
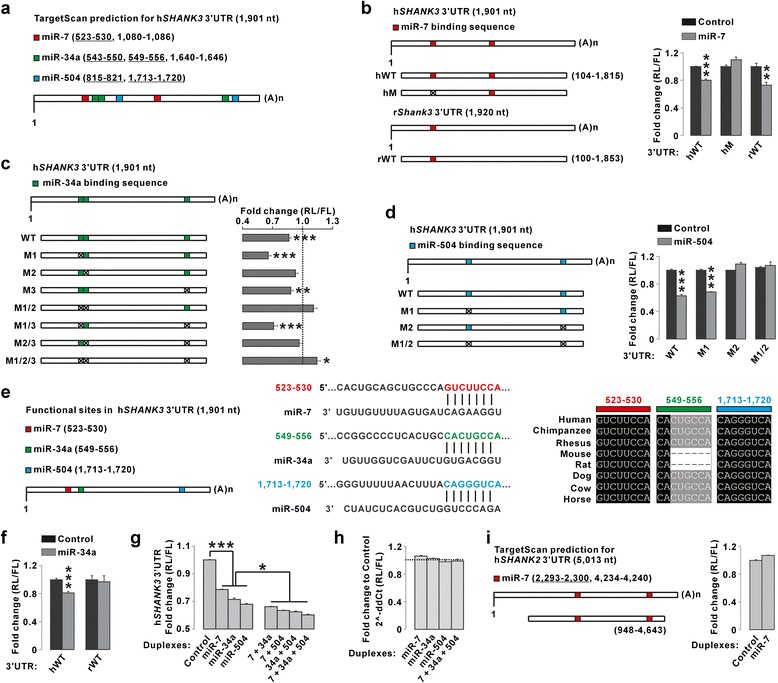
Validation of miR-7, miR-34a, and miR-504 binding sites in the *SHANK3* 3′UTR by luciferase assays in HEK293T cells. **a** TargetScan prediction of the miRNA binding sites in the human *SHANK3* 3′UTR. The underlines indicate evolutionarily conserved binding sites. **b** miR-7 decreased the luciferase activity of the wild-type (hWT), but not the first binding site-mutant (hM), *SHANK3* 3′UTR. The luciferase activity of the rat *Shank3* 3′UTR (rWT), containing only the first binding site, was still reduced by miR-7. RL, Renilla luciferase; FL, firefly luciferase. **c** Mutation of the second miR-34a binding site (M2) blocked its repressive effect on the expression of the *SHANK3* 3′UTR. **d** Mutation of the second miR-504 binding site (M2) blocked its repressive effect on the expression of the *SHANK3* 3′UTR. **e** The validated functional miRNA binding sites in the *SHANK3* 3′UTR (*left*), and their base-pairings with the three miRNAs (*middle*). The sequence alignment across species for each miRNA binding site (*right*). **f** miR-34a did not affect the expression of the rat *Shank3* 3′UTR. **g** miR-7, miR-34a, and miR-504 synergistically decreased the expression of the *SHANK3* 3′UTR. **h** miR-7, miR-34a, and miR-504 did not affect the mRNA levels of the *SHANK3* 3′UTR. **i** miR-7 did not affect the expression of the human *SHANK2* 3′UTR that contains two putative miR-7 binding sites. All data are presented as mean ± SEM. Statistical analyses are in Additional file 1: Table S3

Including poorly conserved binding sites, two to three binding sites in the *SHANK3* 3′UTR for each of the three miRNAs were predicted by TargetScan (Fig. 1a). To validate these binding sites, we performed luciferase assays using constructs containing the *SHANK3* 3′UTR with or without mutations for the binding sites (Fig. 1b–d). Expression of miR-7 decreased the luciferase activity of the wild-type *SHANK3* 3′UTR construct in HEK293T cells, indicating that miR-7 inhibited its expression (Fig. 1b). Between the two putative miR-7 binding sites in the *SHANK3* 3′UTR (523–530 and 1, 080–1, 086), mutation of the first (523–530) abolished the effect of miR-7 on luciferase activity (Fig. 1b). Furthermore, the expression of rat *Shank3* 3′UTR, where the second miR-7 binding site of human 3′UTR is not conserved, was still reduced by miR-7 in the luciferase assay (Fig. 1b), indicating that the first site in the *SHANK3* 3′UTR is the only functional target site for miR-7. Expression of miR-34a or miR-504 also decreased the luciferase activity of the wild-type *SHANK3* 3′UTR construct in HEK293T cells (Fig. 1c and d). Additional luciferase assays using the *SHANK3* 3′UTR constructs with single or multiple mutations in the putative binding sites revealed that each miRNA had a single functional target site in the *SHANK3* 3′UTR (549–556 for miR-34a, and 1, 713-1, 720 for miR-504) (Fig. 1c and d).

The validated target sites for miR-7, miR-34a, and miR-504 were highly conserved across different species (Fig. 1e). However, the miR-34a target site was not conserved in mouse and rat, and consistently, the expression of miR-34a did not affect the luciferase activity of the rat *Shank3* 3′UTR construct in HEK293T cells (Fig. 1f). When expressed at a fixed total amount, miR-7, miR-34a, and miR-504 in combination decreased the luciferase activity of *SHANK3* 3′UTR more efficiently than each miRNA alone, suggesting their synergistic effect (Fig. 1g). The three miRNAs, either alone or together, did not change the mRNA levels of the *SHANK3* 3′UTR luciferase construct (Fig. 1h). We also investigated whether the three miRNAs could regulate the expression of *SHANK1* or *SHANK2*, the two other members in the *SHANK* gene family. TargetScan predicted that the *SHANK2* 3′UTR contained conserved miR-7, but not miR-34a or miR-504, binding sites. However, miR-7 did not change the expression of *SHANK2* 3′UTR in luciferase assays (Fig. 1i), which might be, at least partly, due to the difference in the secondary structures of *SHANK3* and *SHANK2* 3′UTRs (Additional file 1: Figure S1). Together, these results suggest that miR-7, miR-34a, and miR-504 directly bind to the *SHANK3* 3′UTR and downregulate its expression.

### miR-7, miR-34a, and miR-504 potentially regulate other targets in the Shank3 interactome

It is not uncommon for a single miRNA to regulate the expression of multiple proteins participating in the same pathway or interacting with each other [36]. Previously, we generated a comprehensive Shank3 protein interactome by combining yeast two-hybrid screening with mouse brain in vivo immunoprecipitation followed by mass-spectrometry analysis [12]. Therefore, we searched for proteins in the Shank3 interactome that might also be regulated by miR-7, miR-34a, or miR-504.

**Fig. 2:**
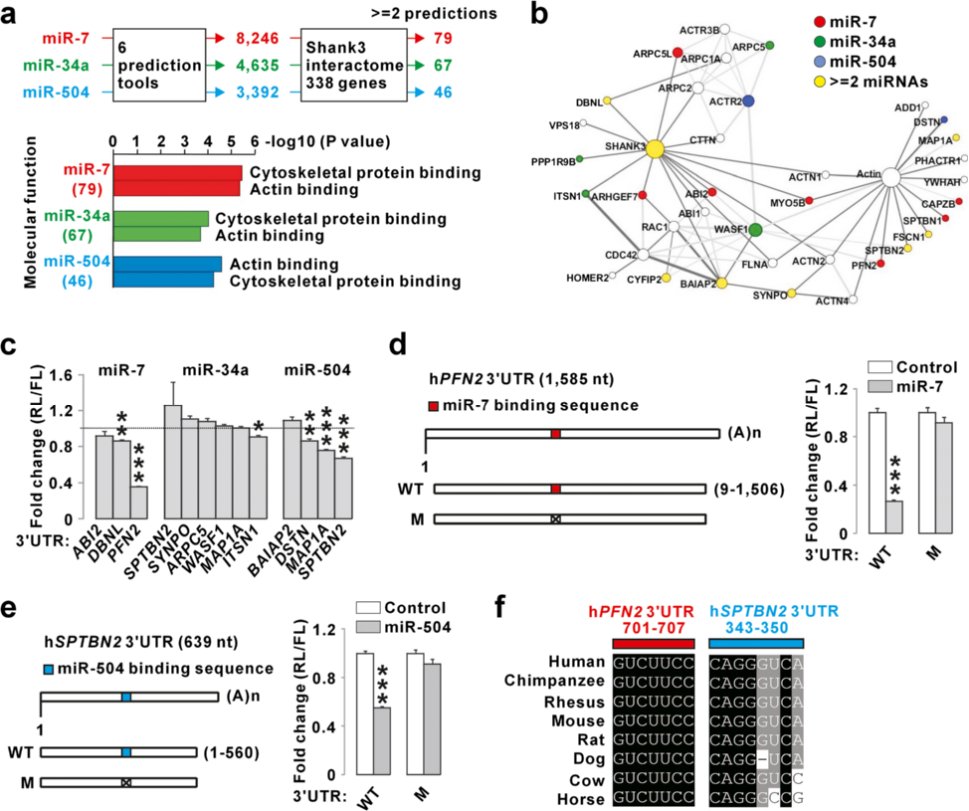
Regulation of Shank3-interacting actin-related proteins by miR-7, miR-34a, and miR-504. **a** Summary of the process identifying putative miR-7, miR-34a, and miR-504 targets in the Shank3 interactome (*top*), and the GO analysis of the putative targets (*bottom*). **b** Actin-related subnetwork of the Shank3 interactome with color codes for the putative miRNA targets. **c** miR-7, miR-34a, and miR-504 decreased the expression of the 3′UTRs of some Shank3-interacting proteins in luciferase assays. **d** Mutation of the miR-7 binding site blocked its repressive effect on the expression of the human *PFN2* 3′UTR. **e** Mutation of the miR-504 binding site blocked its repressive effect on the expression of the human *SPTBN2* 3′UTR. **f** The sequence alignments across species for the miR-7 and miR-34a binding sites in the *PFN2* and *SPTBN2* 3′UTRs, respectively. All data are presented as mean ± SEM. Statistical analyses are in Additional file 1: Table S3

We first pooled the putative human target genes for each of the three miRNAs from six prediction tools (total 8,246 targets for miR-7, 4,635 for miR-34a, and 3,392 for miR-504), and then compared these lists with 338 Shank3 interactome genes [12]. When we picked those targets suggested by more than two prediction tools, 79, 67, and 46 genes in the Shank3 interactome were revealed as putative targets for miR-7, miR-34a, and miR-504, respectively (Fig. 2a). Gene ontology (GO) analysis of these genes suggested actin and cytoskeletal protein binding as the major molecular function (Fig. 2a). Indeed, many genes in the actin-related subnetwork of the Shank3 interactome [12] were predicted as putative targets for the miRNAs (Fig. 2b). To validate these actin-related targets, we first narrowed the gene list down to 11 genes (13 gene-miRNA pairs) which were predicted by more than three different tools. We performed luciferase assays in HEK293T cells with constructs containing the 3′UTRs of the genes, and found that 6 of the 13 gene-miRNA pairs showed decreased luciferase activity after miRNA overexpression (Fig. 2c). We further mutated the putative miRNA binding sites in the 3′UTRs of *PFN2* and *SPTBN2*, the two genes most downregulated by miR-7 and miR-504, respectively, and found that the mutations abolished the effect of the miRNAs in the luciferase assays (Fig. 2d and e). The validated target sites in the *PFN2* and *SPTBN2* 3′UTRs were highly conserved across different species (Fig. 2f). These results suggest that miR-7, miR-34a, and miR-504 could also potentially regulate the expression of some Shank3-interacting proteins, especially those involved in actin regulation.

### miR-7 and miR-504 regulate dendritic spines of cultured hippocampal neurons

**Fig. 3:**
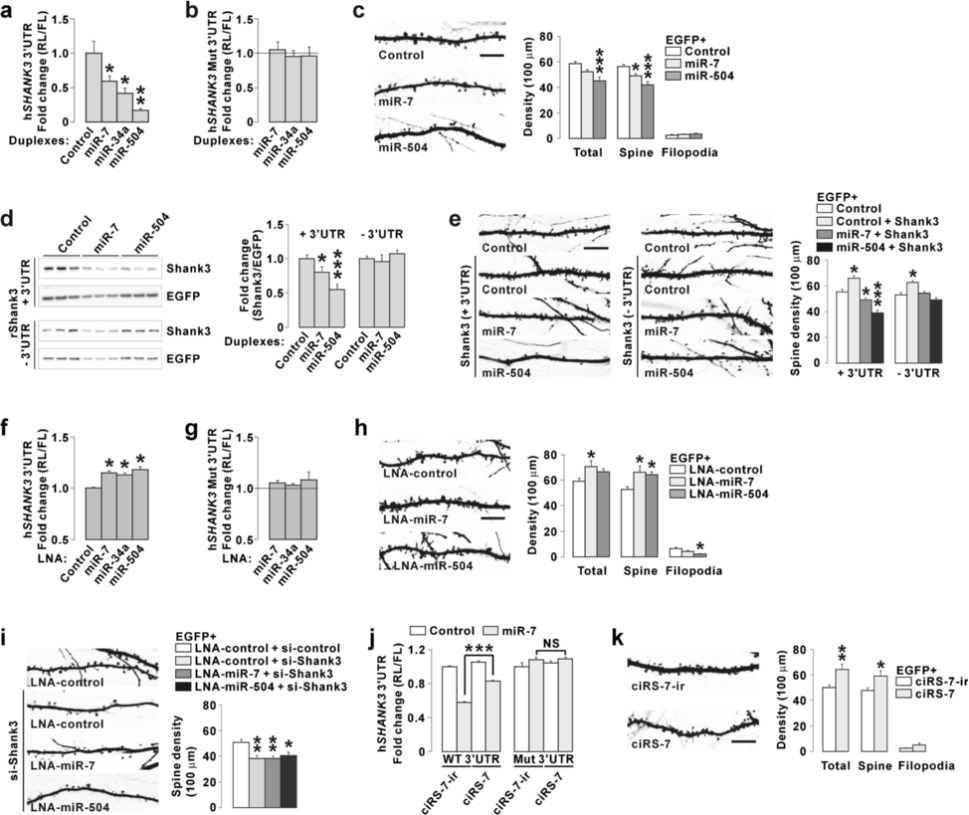
miR-7 and miR-504 regulate dendritic spines of cultured hippocampal neurons in a Shank3-dependent manner. **a** miR-7, miR-34a, or miR-504 overexpression decreased the luciferase activity of wild-type *SHANK3* 3′UTR in cultured mouse hippocampal neurons. **b** miR-7, miR-34a, and miR-504 did not affect the luciferase activity of the mutant *SHANK3* 3′UTR constructs in cultured neurons. **c** Overexpression of miR-7 or miR-504 decreased the density of dendritic spines in cultured mouse hippocampal neurons (*n* = 16–30). **d** Representative western blot images show that overexpression of miR-7 or miR-504 decreased the expression of the rat Shank3 construct with the 3′UTR, but not of that without the 3′UTR, in HEK293T cells. EGFP expressing plasmid was co-transfected as an internal control. Notably, we observed decreased expression of EGFP when miR-7 was cotransfected. Nevertheless, after normalization with EGFP, the expression of Shank3 with 3′UTR, but not that without 3′UTR, was significantly decreased by miR-7. **e** Co-transfection of the Shank3 construct without the 3′UTR, but not that with the 3′UTR, rescued the decreased dendritic spine density by miR-7 or miR-504 overexpression (*n* = 20–30). **f** LNA-inhibitor against miR-7, miR-34a, or miR-504 increased the expression of co-transfected *SHANK3* 3′UTR in cultured mouse hippocampal neurons. **g** LNA-inhibitor against miR-7, miR-34a, and miR-504 did not affect the expression of the mutant *SHANK3* 3′UTR in cultured neurons. **h** LNA-inhibitor against miR-7 or miR-504 increased dendritic spines in cultured neurons (*n* = 18–20). **i** Co-transfection of *Shank3* siRNA blocked the increase of dendritic spines in response to miR-7 or miR-504 inhibition (*n* = 16–20). **j** ciRS-7 partially blocked the repressive effect of miR-7 on the expression of wild-type *SHANK3* 3′UTR in HEK293T cells. Neither miR-7 nor ciRS-7 changed the expression of miR-7 binding-mutant *SHANK3* 3′UTR. ciRS-7-ir is a control plasmid that does not express the circular RNA. **k** Overexpression of ciRS-7 increased dendritic spines in cultured mouse hippocampal neurons (*n* = 15–20). Scale bar, 10 μm. All data are presented as mean ± SEM. Statistical analyses are in Additional file 1: Table S3

Based on the results from HEK293T cells, we investigated the roles of the three miRNAs in neurons. We first repeated the luciferase assays in cultured mouse hippocampal neurons by co-transfecting the miRNAs and luciferase constructs. Consistent with the results from HEK293T cells (Fig. 1), miR-7, miR-34a, and miR-504 decreased the luciferase activity of the wild-type, but not the respective binding site-mutant, *SHANK3* 3′UTR constructs (Fig. 3a and b).

Shank3 overexpression increases while its knockdown decreases the number of dendritic spines in cultured neurons [14, 37]. Thus, we tested whether the miRNAs targeting *SHANK3* 3′UTR could also affect dendritic spines. We transfected enhanced green fluorescent protein (EGFP)-expressing plasmid with control miRNA, miR-7, or miR-504 into cultured mouse hippocampal neurons, and immunostained the neurons with GFP antibody to visualize dendritic protrusions. We did not include miR-34a in the experiment because there is no miR-34a target sequence in the mouse *Shank3* 3′UTR (Fig. 1e and f). However, overexpression of miR-34a was shown to alter dendritic protrusions in cultured mouse neurons by regulating the expression of other synaptic proteins [38]. We found that both miR-7 and miR-504 overexpression decreased dendritic spine density, but neither of the miRNAs affected filopodia (Fig. 3c). To understand whether the spine changes were due to the miRNA-mediated decrease in Shank3 expression, we designed rescue experiments using the full-length rat Shank3-expressing constructs with or without the 3′UTR. We reasoned that only the construct without 3′UTR could rescue the spine phenotype, as it has no miRNA binding site and thus resistant to miRNA overexpression. Indeed, in HEK293T cells, miR-7 and miR-504 reduced the expression of Shank3 proteins from the construct with the 3′UTR, but did not affect the construct without the 3′UTR (Fig. 3d). We transfected cultured hippocampal neurons with control miRNA, miR-7, or miR-504 in combination with the two Shank3 constructs. When co-transfected with control miRNA, Shank3 expression, regardless of the presence of the 3′UTR, caused a significantly higher dendritic spine density compared to that observed with EGFP expression alone (Fig. 3e). However, only the Shank3 construct without the 3′UTR could rescue the decreased spine density in response to miR-7 or miR-504 overexpression (Fig. 3e). Consistently, the expression of Shank3 construct with 3′UTR, but not that without 3′UTR, was decreased by miR-7 and miR-504 in cultured neurons measured by immunostaining (Additional file 1: Figure S2).

As miR-7, miR-34a, and miR-504 are expressed in mouse hippocampal neurons [39–41], we decided to test the effect of inhibition of endogenous miRNAs on *SHANK3* expression and dendritic spines. Transfection of locked-nucleic acid (LNA) inhibitors against each of the miRNAs increased the luciferase activity of wild-type, but not the respective binding site-mutant, *SHANK3* 3′UTR constructs, suggesting that endogenous miR-7, miR-34a, and miR-504 could regulate *SHANK3* expression (Fig. 3f and g). Moreover, opposite to the miRNA overexpression, miR-7 or miR-504 inhibition increased spine density in cultured mouse hippocampal neurons (Fig. 3h). In the case of miR-504 inhibition, the density of the filopodia was decreased (Fig. 3h). To understand whether Shank3 expression was required for the spine changes observed after miR-7 or miR-504 inhibition, we co-transfected a previously validated siRNA targeting mouse *Shank3* [12]. We found that *Shank3* siRNA alone resulted in lower spine density compared to that observed with control siRNA, and that it also blocked the increase in spine density in response to miR-7 or miR-504 inhibition (Fig. 3i).

Recently, an endogenous circular RNA that has more than 70 binding sites for miR-7 was identified [42, 43]. This circular RNA can indirectly increase the expression of other miR-7 targets by sequestering miR-7, and was thus named as a circular RNA sponge for miR-7 (ciRS-7) [42]. Indeed, in HEK293T cells, ciRS-7 expression partially blocked the inhibitory effect of miR-7 on the luciferase activity of the wild-type, but not the miR-7 binding-mutant *SHANK3* 3′UTR construct (Fig. 3j). As ciRS-7 and miR-7 were detected in neuronal tissues including the hippocampus [42, 43], we examined the effect of ciRS-7 overexpression on dendritic spines. We found that cultured hippocampal neurons transfected with ciRS-7 showed increased spine density compared to the neurons transfected with control plasmid (ciRS-7-ir) (Fig. 3k), which was the same phenotype as that observed in response to a miR-7 LNA inhibitor (Fig. 3h). Taken together, these results suggest that miR-7, miR-34a, and miR-504 could regulate *SHANK3* expression in cultured mouse hippocampal neurons, and that miR-7 and miR-504 could regulate dendritic spines in a Shank3-dependent manner.

### Human and mouse brain expression patterns of miR-504

**Fig. 4:**
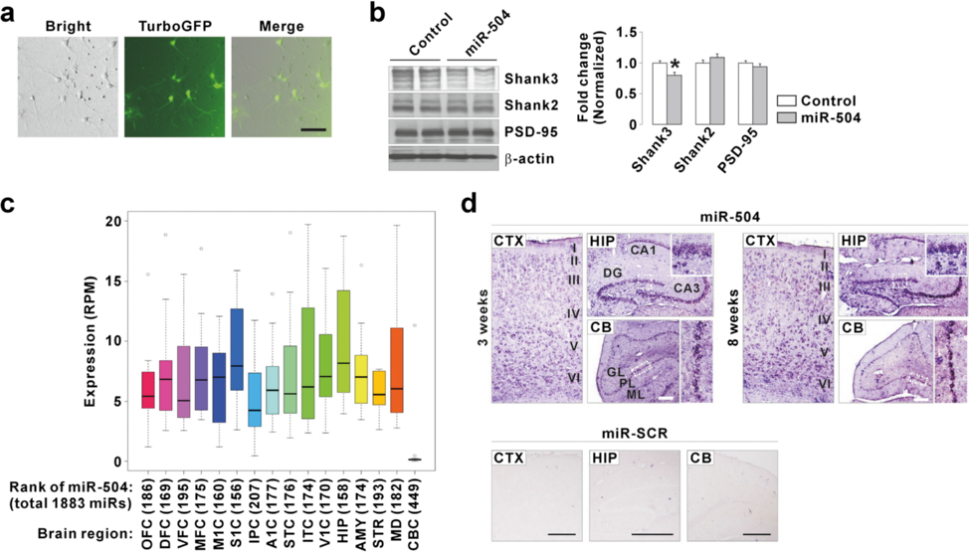
Lentiviral overexpression of miR-504 in cultured neurons, and expression analysis of miR-504 in human and mouse brain. **a** Representative images of live neurons infected with lentivirus expressing TurboGFP together with miR-504. Scale bar, 100 μm. **b** Representative western blot images and quantification showing that lentiviral overexpression of miR-504 decreased endogenous Shank3, but not Shank2 and PSD-95, proteins in cultured hippocampal neurons. **c** Box plots showing the expression distribution of miR-504 in 16 human brain regions. The black line in each box indicates the median value. The lower and upper hinges of each box indicate the lower and upper quartile values, respectively. The whiskers of each box indicate the most extreme data values within 1.5 times of the interquartile range. The open circles indicate the data values beyond the whisker limits. The number in parentheses associated with each brain region is the expression rank of miR-504 among all the 1,883 miRNAs available for the brain region. OFC, orbital prefrontal cortex; DFC, dorsolateral prefrontal cortex; VFC, ventrolateral prefrontal cortex; MFC, medial prefrontal cortex; M1C, primary motor (M1) cortex; S1C, primary somatosensory (S1) cortex; IPC, posterior inferior parietal cortex; A1C, primary auditory (A1) cortex; STC, superior temporal cortex; ITC, inferior temporal cortex; V1C, primary visual (V1) cortex; HIP, hippocampus; AMY, amygdala; STR, striatum; MD, mediodorsal nucleus of the thalamus; CBC, cerebellar cortex. **d** *In situ* hybridization of miR-504 in mouse brains. miR-504 signals were detected in neurons of cortex and hippocampus, and in Purkinje cells of cerebellum from 3- and 8-week-old mice. The probe against scrambled miRNA (miR-SCR) was used as a negative control. CB, cerebellum; CTX, cortex; DG, dentate gyrus; GL, granular layer; ML, molecular layer; PL, Purkinje cell layer. Scale bar, 500 μm. All data are presented as mean ± SEM. Statistical analyses are in Additional file 1: Table S3

We decided to further characterize miR-504, as it most significantly downregulated *SHANK3* levels and changed dendritic spines among the three miRNAs we tested (Figs. 1 and 3). Notably, expression of miR-504 was shown to be increased in the dorsolateral prefrontal cortex from postmortem brains of bipolar disorder [32], and in the nucleus accumbens in a rat model of depression induced by maternal deprivation followed by chronic unpredictable stress [44]. These reports, together with our results from spine analysis, prompted us to test the effect of miR-504 overexpression on endogenous Shank3 levels. We used a lentivirus expressing TurboGFP together with control miRNA or miR-504. Consistent with our results from the luciferase assays, infection of the virus expressing miR-504 decreased Shank3 proteins in cultured hippocampal neurons confirmed by western blot experiments (Fig. 4a and b). The expression of other synaptic proteins, Shank2 and PSD-95, were not significantly changed by miR-504 (Fig. 4b).

Although miR-504 was detected in some brain regions including the cortex [32], hippocampus [41], and nucleus accumbens [44], its overall expression pattern in the brain has not been characterized. To understand this, we first investigated the regional expression pattern of miR-504 in human brain by performing bioinformatic analyses on the developing human brain miRNA sequencing data from the BrainSpan database (http://www.brainspan.org). We found that miR-504 was detected throughout all brain regions, with the highest median value of reads per million mapped reads (RPM) found in the hippocampus (HIP), and the lowest median value found in the cerebellar cortex (CBC) (Fig. 4c). In terms of the expression rank of miR-504 among all the 1,883 miRNAs available for each brain region, the primary somatosensory cortex (S1C) and cerebellar cortex showed the highest and lowest values, respectively (Fig. 4c). To directly visualize miR-504 expression, we next performed *in situ* hybridization of miR-504 in the brains of 3- and 8-week-old mice. Similar to the expression pattern in human brains, strong miR-504 signals were detected in the cortex and hippocampus from both stages (Fig. 4d). In the cerebellum, the signal was mainly detected in the Purkinje cells (Fig. 4d). Together, these results suggest that the increased expression of miR-504 observed in postmortem brains of bipolar disorder and in a rat model of depression, might possibly lead to a decrease in Shank3 protein in some brain regions.

## Discussion

Fine-tuning of dosage-sensitive *SHANK3* should require tight control of the entire process of gene expression, from transcription to post-translational modification. Therefore, it is possible that abnormalities in the regulatory processes of *SHANK3* expression result in pathogenic outcomes similar to those observed with genetic mutations of *SHANK3* itself. One such example is the DNA methylation of *SHANK3*, which is critical for tissue-specific control of *SHANK3* expression [45–47]. Recently, Zhu et al. found that DNA methylation of the intragenic CpG islands of *SHANK3* was significantly increased in postmortem brain tissues of ASD patients, which could be associated with altered expression and alternative splicing of *SHANK3* in ASD brains [48]. In the present study, we tried to propose and test an additional example by investigating miRNA-dependent regulation of *SHANK3* expression. We found that miR-7, miR-34a, and miR-504, three miRNAs with altered expression profiles in multiple neuropsychiatric disorders, directly regulate *SHANK3* expression. Moreover, these miRNAs potentially regulate other targets interacting with Shank3 in actin-regulatory pathway. Consistently, both gain- and loss-of-function experiments showed that miR-7 and miR-504 control actin-rich dendritic spines. Importantly, these dendritic spine changes were dependent on Shank3, suggesting that Shank3 functions as a core protein among the actin-related miR-7 and miR-504 targets in regulating dendritic spines. Together, our results provide new insight into the miRNA-mediated regulation of *SHANK3* expression, and its potential implications for multiple neuropsychiatric disorders with altered expression profiles of miR-7, miR-34a, and miR-504.

According to the recent study from Wang et al. there are at least ten Shank3 isoforms from the usage of intragenic promoters and alternative splicing [49]. Although each isoform has brain region- and development-specific expression pattern, six out of ten major Shank3 isoforms have sterile alpha motif (SAM) domain at the C-terminus, which is encoded by the last exon containing the 3′UTR sequence. Moreover, using 3′-end sequencing and identifying polyA signal hexamers, Epstein et al. showed that *Shank3* 3′UTR has only one polyadenylation site, whereas *Shank1* and *Shank2* 3′UTR could have more than one polyadenylation sites [50]. Together, these results suggest that miR-7, miR-34a, and miR-504 could commonly regulate the expression of at least six major Shank3 isoforms (a, a[E10-12S V], c, d, e, and f) (Additional file 1: Figure S3) [8, 49]. However, the in vivo effects of miRNAs on Shank3-related brain function could be various, because of the specific spatiotemporal expression of each miRNA. The combinatorial analysis on the brain expressions of *SHANK3* isoforms and the miRNAs targeting *SHANK3* will help us better understand this regulatory process. Furthermore, other targets of the miRNAs and their roles in synaptic function need to be more characterized.

Among the three miRNAs, we further characterized the most potent miR-504. It is tempting to speculate that increased expression of miR-504 in the prefrontal cortex of bipolar disorder [32] might contribute to pathogenesis, at least partly, by downregulating *SHANK3* expression and dendritic spines as we demonstrated in the cultured hippocampal neurons. Indeed, deletions of *SHANK3* gene have been identified in some patients diagnosed with bipolar disorder [51–53]. Bipolar disorder is a devastating mental illness that causes recurrent mood swings between depression and mania. Although the detailed pathophysiology of bipolar disorder remains largely unknown, both decreased and elevated activity of the prefrontal cortex have been detected in patients [54], suggesting that precise control of neuronal activity is critical for preventing bipolar disorder. Supporting this idea, we also reported manic-like behavior in the transgenic mice overexpressing *Shank3* as well as in the patients with *SHANK3* duplications [12, 55]. Therefore, both deletions and duplications of *SHANK3* could be associated with bipolar disorder by disturbing neuronal homeostasis.

In addition to *SHANK3*, miR-504 also regulates the expression of the dopamine D1 receptor gene (*DRD1*) [56], expression or activity of which is associated with multiple neuropsychiatric disorders including mood disorders [57]. Moreover, maternal deprivation followed by chronic unpredictable stress increases miR-504 expression in the rat nucleus accumbens where the miR-504 levels are positively correlated with the severity of depression-like behavior after stress [44]. These results suggest that miR-504 might be an important regulator of gene expression in some cortical and limbic regions associated with mood disorders, an idea that is also partly supported by our results for the human and mouse brain distribution of miR-504. Generation and behavioral characterization of animal models with altered miR-504 expression in these brain regions could help us test this intriguing hypothesis.

## Conclusions

In this study, we showed post-transcriptional regulation of *SHANK3* expression in neurons by three miRNAs, miR-7, miR-34a, and miR-504. We propose that similar approaches could be applied to other dosage-sensitive genes and miRNAs commonly associated with some neuropsychiatric disorders, which could provide new insights into the molecular basis of pathophysiology, and potentially into novel diagnostic and therapeutic approaches.

## Methods

### Luciferase assay

The 3′UTR regions of human *SHANK3* (NM_033517.1, 104-1,815), rat *Shank3* (NM_021676.1, 100-1, 853), and human *ABI2* (NM_005759.5, 1-1, 036), *ARPC5* (NM_005717.3, 371-1, 184), *BAIAP2* (NM_017450.2, 1-602), *DBNL* (NM_001122956.1, 31-782), *DSTN* (NM_001011546.1, 1-703), *ITSN1* (NM_001001132.1, 1-897), *MAP1A* (NM_002373.5, 55-1,386), *PFN2* (NM_053024.3, 9-1,506), *SHANK2* (NM_012309.3, 948-4,643), *SPTBN2* (NM_006946.2, 1-555), *SYNPO* (NM_007286.5, 1, 238-2090), and *WASF1* (NM_001024934.1, 41-643) were PCR amplified from brain cDNA libraries and subcloned into the psiCHECK-2 vector (Promega). Mutagenesis reactions of the 3′UTR constructs were performed using the QuikChange XL Site-Directed Mutagenesis Kit (Agilent Technologies) to change the three nucleotides of the miRNA seed match regions (position 4 to 6) into complementary sequences. HEK293T cells in 24-well plates were transfected with 30 ng of psiCHECK-2 construct plus 20 pmol of miRNA duplex (miRIDIAN Dharmacon) using Lipofectamine 2000 (Invitrogen). To test the effect of a circular RNA sponge for miR-7, 200 ng of ciRS-7-ir or ciRS-7 plasmids (kindly gifted from Dr. Jorgen Kjems) were co-transfected with 30 ng of psiCHECK-2 *SHANK3* 3′UTR and 6 pmol of miR-7 duplex. After 24 h, luciferase activities were measured using the Dual Luciferase Reporter Assay System (Promega).

### RNA extraction and quantitative real-time reverse transcription PCR

Total RNA was extracted from HEK293T cells in 24-well plates using a miRNeasy minikit (Qiagen) according to the manufacturer’s instructions. 1 μg of DNase-treated total RNA was used to synthesize cDNA using the Quantitect Reverse Transcription Kit (Qiagen). Quantitative real-time reverse transcription PCR (qRT-PCR) experiments were performed using the CFX96 Touch Real-Time PCR Detection System (Bio-Rad Laboratories) with PerfeCta SYBR Green FastMix, ROX (Quanta Biosciences). The primers for the qRT-PCR reactions are as follows:

*Renilla* luciferase gene forward 5′-CGAAGAGGGCGAGAAAATGG-3′

reverse 5′-ACTCCTCAGGCTCCAGTTTC-3′

firefly luciferase gene forward 5′-GCATTTCTCAGCCTACCGTG-3′

reverse 5′-CAGCTTCTTCTGCACGTTCA-3′

### Bioinformatics

The secondary structures of *SHANK3* and *SHANK2* 3′UTRs were predicted by *RNAfold* (http://rna.tbi.univie.ac.at/cgi-bin/RNAfold.cgi). To identify the putative miR-7, miR-34a, and miR-504 targets in the Shank3 interactome, we first pooled all the putative targets for each miRNA from six different prediction tools (TargetScan [http://targetscan.org/], MiRANDA [http://www.microrna.org/microrna/home.do], PicTar2 [http://pictar.mdc-berlin.de/], DIANA-microT [http://diana.cslab.ece.ntua.gr/microT/], miRDB [http://mirdb.org/miRDB/], and EIMMo3 [http://www.mirz.unibas.ch/ElMMo3/]). These lists (8,246 targets for miR-7, 4,635 for miR-34a, and 3,392 for miR-504) were compared with the 388 proteins in the Shank3 interactome. The targets predicted by more than two different prediction tools were further considered for gene ontology (GO) analysis using DAVID software (version 6.7) (https://david.ncifcrf.gov/).

To understand the human brain distribution of miR-504, the miRNA sequencing data of the developing human brain were downloaded from the BrainSpan database (http://www.brainspan.org). This dataset contains 216 samples spatially covering 16 brain regions and temporally spanning developmental periods from 4 months to 23 years of age. Reads were normalized to reads per million mapped reads (RPM) using the formula:

$$ R=\frac{10^6C}{NL} $$

where C is the number of reads mapped to one miRNA, N is the total number of mapped reads in the sample, and L is the length of the miRNA. To explore the regional expression pattern of miR-504, the dataset was divided into 16 brain regions, with the samples from the same brain region being grouped together. To obtain the expression rank of miR-504 among all the 1,883 miRNAs for each brain region, the median value of each miRNA across all the samples in the brain region was computed, and the resulting median values of all miRNAs were ranked in descending order.

### Hippocampal neuron culture, transfection, and immunostaining

Hippocampal neurons were prepared from postnatal day 1 mice of C57BL/6 J background, and cultured as described previously [36]. At 7 days in vitro (DIV 7), neurons on each coverslip in 24-well plates were transfected with 200 ng of pEGFP-C1 (Clontech), 30 pmol of miRNA duplex, and 200 ng of pcDNA3.1 (as filler, Invitrogen) or HA-Shank3 (with or without 3′UTR) plasmids using Lipofectamine 2000. For inhibition of endogenous miRNAs, neurons were transfected with 200 ng of pEGFP-C1, and 50 pmol of LNA-inhibitor (miRCURY EXIQON) or 800 ng of ciRS-7-ir/ciRS-7 plasmids. 30 pmol of *Shank3* siRNA (Ambion, s81603) was co-transfected for the rescue experiments. At DIV 14, transfected neurons were fixed with 4 % formaldehyde/4 % sucrose in phosphate-buffered saline (PBS), and permeabilized with 0.2 % Tx-100 in PBS. PBS with 0.1 % BSA and 3 % horse serum was used for blocking and antibody incubation. Rabbit GFP (1:500, Abcam, ab290) and mouse HA (1:300, Santa Cruz) antibodies were used for immunostaining.

### Lentiviral infection of cultured hippocampal neurons

Lentiviral particles expressing TurboGFP together with the mature form of a control miR (Dharmacon, S05-005000-01) or miR-504 (shMIMIC Dharmacon, VSM6213-213638367) under a CAG promoter were applied to cultured hippocampal neurons (DIV 1, multiplicity of infection of 5) prepared from embryonic day 18 rats. At DIV 14, neurons were processed for western blot experiments.

### Western blot and antibodies

HEK293T cells were briefly washed with ice-cold PBS and lysed in RIPA buffer (25 mM Tris–HCl pH 7.6, 150 mM NaCl, 1 % NP-40, 1 % sodium deoxycholate, 0.1 % SDS) with freshly added protease inhibitor cocktail (Roche). After 20 min incubation on ice, the lysates were centrifuged at 12,000 g for 20 min. The proteins in the supernatant were quantified and boiled with Laemmli sample buffer. For cultured neurons, 2x Laemmli sample buffer was directly applied after a brief wash with ice-cold PBS. Rabbit Shank3 (1:1000, Santa Cruz, H-160), rabbit Shank2 (1:500, #1136) [58], mouse PSD-95 (1:1000, NeuroMab, K28/43), and mouse β-actin (1:5000, Abcam, ab6276) antibodies were used for western blot experiments.

### *In situ* hybridization

The 3 and 8 week-old male C57BL/6 mice were deeply anesthetized, and then perfused with PBS, followed by 4 % paraformaldehyde (PFA) in PBS. Subsequently, isolated brains were post-fixed in 4 % PFA overnight, and then cryoprotected in 30 % sucrose in PBS. Brains were sectioned (14 μm thickness) on slide glasses, and then air-dried. The sections were fixed with 4 % PFA for 20 min and subsequently washed with PBS. The acetylation reaction (with 0.25 % acetic anhydride in 0.1 M RNase-free triethanolamine-HCl, pH 8.0) was performed for 10 min at room temperature (RT). After washing with PBS, the brain sections were incubated in hybridization buffer for 2 h at 60 °C, followed by overnight hybridization with 60 ng/ml DIG-labeled riboprobe against miR-504 (Exiqon, 38654-15) at 60 °C. On the next day, the brain sections were washed with buffers in the following order: 50 % formamide, 0.2X saline sodium citrate (SSC), PBST (0.1 % Tx-100 in 0.1 M PBS), 0.2X SSC, and PBST at 60 °C. After blocking with 10 % sheep serum in PBST for 1 h, anti-DIG-AP (1:2000; Roche) was applied to the brain sections overnight at RT. After washing with PBST and PBS, brain slices were incubated in nitro blue tetrazolium/5-bromo-4-chloro-3-indolyl-phosphate (NBT/BCIP) developing solution for 8 h to overnight. To stop the reaction, brain sections were incubated in tap water. After dehydration, brain sections were air-dried, incubated with xylene, and mounted with Permount (Fisher Scientific). Images were acquired with BX53F microscope (Olympus).

### Image acquisition and statistical analysis

For western blot experiments, images were acquired either with an LAS 4000 (GE Healthcare) or by developing on films followed by scanning, and quantified with an ImageJ software package. For immunostaining of cultured neurons, all z-stack images were acquired with a LSM780 (Zeiss) confocal microscope under the same parameter settings, and dendritic protrusions were analyzed using ImageJ. The dendritic spines were defined as protrusions with heads, and filopodia were defined as protrusions without head and having a length at least twice the width. All data were presented as mean ± SEM. Statistical significance was determined by Student’s *t*-test or one-way ANOVA with post hoc Tukey’s multiple comparisons using GraphPad Prism 6. *P < 0.05, **P < 0.01, ***P < 0.001.

## Additional file

Additional file 1: Table S1. The putative miRNA families targeting the *SHANK3* 3′UTR predicted by TargetScan (release 6.2). The number of binding sites for each miRNA family, and the binding type (8mer, 7mer-m8, or 7mer-1A) are described. **Table S2**. Altered expression profiles of miR-7, miR-34a, and miR-504 in multiple neuropsychiatric disorders. **Table S3**. Summary of statistical analyses for the experiments. **Figure S1**. The secondary structures of *SHANK3* (a) and *SHANK2* (b) 3′UTRs predicted by RNAfold (http://rna.tbi.univie.ac.at/cgi-bin/RNAfold.cgi) according to the minimum free energy. The color code represents base-pair probabilities. **Figure S2**. The expression of Shank3 construct with 3′UTR, but not that without 3′UTR, was significantly decreased by miR-7 and miR-504 in mouse cultured neurons. Statistical analyses are in Additional file 1: Table S3. **Figure S3**. The ten major Shank3 isoforms (Wang et al. Molecular Autism 2014, 5:30) (a), and the nucleotide sequence of the last exon (exon 22) of *Shank3* gene (b). The Shank3 isoforms containing SAM domain at the C-terminus are labeled with red color. (DOCX 3382 kb)

## Abbreviations

A1C: primary auditory A1 cortex
AMY: Amygdale
ASDs: autism spectrum disorders
ciRS-7: circular RNA sponge for miR-7
CBC: cerebellar cortex
CNVs: copy number variations
DFC: dorsolateral prefrontal cortex
DG: dentate gyrus
GL: granular layer
GO: gene ontology
HIP: hippocampus
IPC: posterior inferior parietal cortex
ITC: inferior temporal cortex
LNA: locked-nucleic acid
MFC: medial prefrontal cortex
M1C: primary motor M1 cortex
miRNAs: microRNAs
MD: mediodorsal nucleus of the thalamus
ML: molecular layer
OFC: orbital prefrontal cortex
PSD: postsynaptic density
PL: purkinje cell layer
S1C: primary somatosensory S1 cortex
SAM: sterile alpha motif
STC: superior temporal cortex
STR: striatum
V1C: primary visual V1 cortex
VFC: ventrolateral prefrontal cortex
