# Supplementary material for: Post-transcriptional regulation of SHANK3 expression by microRNAs related to multiple neuropsychiatric disorders
Source: Mol Brain. 2015 Nov 16;8:74. doi: 10.1186/s13041-015-0165-3 (PMC4647645; doi:10.1186/s13041-015-0165-3)

**Post-transcriptional regulation of *SHANK3* expression by microRNAs related to multiple neuropsychiatric disorders**

Su-Yeon Choi, Kaifang Pang, Joo Yeon Kim, Jae Ryun Ryu, Hyojin Kang, Zhandong Liu, Won-Ki Kim, Woong Sun, Hyun Kim and Kihoon Han

**Table S1** The putative miRNA families targeting the *SHANK3* 3’UTR predicted by TargetScan (release 6.2). The number of binding sites for each miRNA family, and the binding type (8mer, 7mer-m8, or 7mer-1A) are described.

| **miRNA families broadly conserved among vertebrates** | | | | | | | | |
| --- | --- | --- | --- | --- | --- | --- | --- | --- |
| **miRNA** | **Conserved sites** | | | | **Poorly conserved sites** | | | |
|  | **Total** | **8mer** | **7mer-m8** | **7mer-1A** | **Total** | **8mer** | **7mer-m8** | **7mer-1A** |
| [**miR-34ac/34bc-5p/449abc/449c-5p**](http://www.targetscan.org/cgi-bin/vert_61/view_gene.cgi?gs=SHANK3&taxid=9606&members=miR-34ac/34bc-5p/449abc/449c-5p&showcnc=1&shownc=1) | [1](http://www.targetscan.org/cgi-bin/vert_61/view_gene.cgi?gs=SHANK3&taxid=9606&members=miR-34ac/34bc-5p/449abc/449c-5p&showcnc=1&shownc=1) | 1 | 0 | 0 | [1](http://www.targetscan.org/cgi-bin/vert_61/view_gene.cgi?gs=SHANK3&taxid=9606&members=miR-34ac/34bc-5p/449abc/449c-5p&showcnc=1&shownc=1) | 0 | 0 | 1 |
| [miR-10abc/10a-5p](http://www.targetscan.org/cgi-bin/vert_61/view_gene.cgi?gs=SHANK3&taxid=9606&members=miR-10abc/10a-5p&showcnc=1&shownc=1) | [2](http://www.targetscan.org/cgi-bin/vert_61/view_gene.cgi?gs=SHANK3&taxid=9606&members=miR-10abc/10a-5p&showcnc=1&shownc=1) | 0 | 2 | 0 | 0 | 0 | 0 | 0 |
| [miR-191](http://www.targetscan.org/cgi-bin/vert_61/view_gene.cgi?gs=SHANK3&taxid=9606&members=miR-191&showcnc=1&shownc=1) | [1](http://www.targetscan.org/cgi-bin/vert_61/view_gene.cgi?gs=SHANK3&taxid=9606&members=miR-191&showcnc=1&shownc=1) | 0 | 1 | 0 | 0 | 0 | 0 | 0 |
| [**miR-7/7ab**](http://www.targetscan.org/cgi-bin/vert_61/view_gene.cgi?gs=SHANK3&taxid=9606&members=miR-7/7ab&showcnc=1&shownc=1) | [1](http://www.targetscan.org/cgi-bin/vert_61/view_gene.cgi?gs=SHANK3&taxid=9606&members=miR-7/7ab&showcnc=1&shownc=1) | 1 | 0 | 0 | [1](http://www.targetscan.org/cgi-bin/vert_61/view_gene.cgi?gs=SHANK3&taxid=9606&members=miR-7/7ab&showcnc=1&shownc=1) | 0 | 0 | 1 |
| [miR-128/128ab](http://www.targetscan.org/cgi-bin/vert_61/view_gene.cgi?gs=SHANK3&taxid=9606&members=miR-128/128ab&showcnc=1&shownc=1) | [1](http://www.targetscan.org/cgi-bin/vert_61/view_gene.cgi?gs=SHANK3&taxid=9606&members=miR-128/128ab&showcnc=1&shownc=1) | 1 | 0 | 0 | 0 | 0 | 0 | 0 |
| [miR-214/761/3619-5p](http://www.targetscan.org/cgi-bin/vert_61/view_gene.cgi?gs=SHANK3&taxid=9606&members=miR-214/761/3619-5p&showcnc=1&shownc=1) | 0 | 0 | 0 | 0 | [2](http://www.targetscan.org/cgi-bin/vert_61/view_gene.cgi?gs=SHANK3&taxid=9606&members=miR-214/761/3619-5p&showcnc=1&shownc=1) | 0 | 2 | 0 |
| [miR-375](http://www.targetscan.org/cgi-bin/vert_61/view_gene.cgi?gs=SHANK3&taxid=9606&members=miR-375&showcnc=1&shownc=1) | [1](http://www.targetscan.org/cgi-bin/vert_61/view_gene.cgi?gs=SHANK3&taxid=9606&members=miR-375&showcnc=1&shownc=1) | 0 | 0 | 1 | 0 | 0 | 0 | 0 |
| [miR-15abc/16/16abc/195/322/424/497/1907](http://www.targetscan.org/cgi-bin/vert_61/view_gene.cgi?gs=SHANK3&taxid=9606&members=miR-15abc/16/16abc/195/322/424/497/1907&showcnc=1&shownc=1) | 0 | 0 | 0 | 0 | [2](http://www.targetscan.org/cgi-bin/vert_61/view_gene.cgi?gs=SHANK3&taxid=9606&members=miR-15abc/16/16abc/195/322/424/497/1907&showcnc=1&shownc=1) | 0 | 2 | 0 |
| [miR-27abc/27a-3p](http://www.targetscan.org/cgi-bin/vert_61/view_gene.cgi?gs=SHANK3&taxid=9606&members=miR-27abc/27a-3p&showcnc=1&shownc=1) | [1](http://www.targetscan.org/cgi-bin/vert_61/view_gene.cgi?gs=SHANK3&taxid=9606&members=miR-27abc/27a-3p&showcnc=1&shownc=1) | 0 | 1 | 0 | 0 | 0 | 0 | 0 |
| [miR-150/5127](http://www.targetscan.org/cgi-bin/vert_61/view_gene.cgi?gs=SHANK3&taxid=9606&members=miR-150/5127&showcnc=1&shownc=1) | [1](http://www.targetscan.org/cgi-bin/vert_61/view_gene.cgi?gs=SHANK3&taxid=9606&members=miR-150/5127&showcnc=1&shownc=1) | 0 | 0 | 1 | 0 | 0 | 0 | 0 |
| [miR-9/9ab](http://www.targetscan.org/cgi-bin/vert_61/view_gene.cgi?gs=SHANK3&taxid=9606&members=miR-9/9ab&showcnc=1&shownc=1) | [1](http://www.targetscan.org/cgi-bin/vert_61/view_gene.cgi?gs=SHANK3&taxid=9606&members=miR-9/9ab&showcnc=1&shownc=1) | 0 | 0 | 1 | 0 | 0 | 0 | 0 |
|  | | | | | | | | |
| **miRNA families conserved only among mammals** | | | | | | | | |
| **miRNA** | **Conserved sites** | | | | **Poorly conserved sites** | | | |
|  | **Total** | **8mer** | **7mer-m8** | **7mer-1A** | **Total** | **8mer** | **7mer-m8** | **7mer-1A** |
| [**miR-504/4725-5p**](http://www.targetscan.org/cgi-bin/vert_61/view_gene.cgi?gs=SHANK3&taxid=9606&members=miR-504/4725-5p&showcnc=1&shownc=1) | [2](http://www.targetscan.org/cgi-bin/vert_61/view_gene.cgi?gs=SHANK3&taxid=9606&members=miR-504/4725-5p&showcnc=1&shownc=1) | 1 | 1 | 0 | 0 | 0 | 0 | 0 |
| [miR-615-3p](http://www.targetscan.org/cgi-bin/vert_61/view_gene.cgi?gs=SHANK3&taxid=9606&members=miR-615-3p&showcnc=1&shownc=1) | [1](http://www.targetscan.org/cgi-bin/vert_61/view_gene.cgi?gs=SHANK3&taxid=9606&members=miR-615-3p&showcnc=1&shownc=1) | 0 | 1 | 0 | [1](http://www.targetscan.org/cgi-bin/vert_61/view_gene.cgi?gs=SHANK3&taxid=9606&members=miR-615-3p&showcnc=1&shownc=1) | 0 | 0 | 1 |
| [miR-491-5p](http://www.targetscan.org/cgi-bin/vert_61/view_gene.cgi?gs=SHANK3&taxid=9606&members=miR-491-5p&showcnc=1&shownc=1) | [1](http://www.targetscan.org/cgi-bin/vert_61/view_gene.cgi?gs=SHANK3&taxid=9606&members=miR-491-5p&showcnc=1&shownc=1) | 0 | 0 | 1 | [2](http://www.targetscan.org/cgi-bin/vert_61/view_gene.cgi?gs=SHANK3&taxid=9606&members=miR-491-5p&showcnc=1&shownc=1) | 0 | 0 | 2 |
| [miR-378/422a/378bcdefhi](http://www.targetscan.org/cgi-bin/vert_61/view_gene.cgi?gs=SHANK3&taxid=9606&members=miR-378/422a/378bcdefhi&showcnc=1&shownc=1) | [1](http://www.targetscan.org/cgi-bin/vert_61/view_gene.cgi?gs=SHANK3&taxid=9606&members=miR-378/422a/378bcdefhi&showcnc=1&shownc=1) | 0 | 1 | 0 | [1](http://www.targetscan.org/cgi-bin/vert_61/view_gene.cgi?gs=SHANK3&taxid=9606&members=miR-378/422a/378bcdefhi&showcnc=1&shownc=1) | 0 | 1 | 0 |
| [miR-374ab](http://www.targetscan.org/cgi-bin/vert_61/view_gene.cgi?gs=SHANK3&taxid=9606&members=miR-374ab&showcnc=1&shownc=1) | [1](http://www.targetscan.org/cgi-bin/vert_61/view_gene.cgi?gs=SHANK3&taxid=9606&members=miR-374ab&showcnc=1&shownc=1) | 1 | 0 | 0 | 0 | 0 | 0 | 0 |
| [miR-376c/741-5p](http://www.targetscan.org/cgi-bin/vert_61/view_gene.cgi?gs=SHANK3&taxid=9606&members=miR-376c/741-5p&showcnc=1&shownc=1) | [1](http://www.targetscan.org/cgi-bin/vert_61/view_gene.cgi?gs=SHANK3&taxid=9606&members=miR-376c/741-5p&showcnc=1&shownc=1) | 0 | 1 | 0 | 0 | 0 | 0 | 0 |
| [miR-134/3118](http://www.targetscan.org/cgi-bin/vert_61/view_gene.cgi?gs=SHANK3&taxid=9606&members=miR-134/3118&showcnc=1&shownc=1) | 0 | 0 | 0 | 0 | [2](http://www.targetscan.org/cgi-bin/vert_61/view_gene.cgi?gs=SHANK3&taxid=9606&members=miR-134/3118&showcnc=1&shownc=1) | 0 | 1 | 1 |
| [miR-494](http://www.targetscan.org/cgi-bin/vert_61/view_gene.cgi?gs=SHANK3&taxid=9606&members=miR-494&showcnc=1&shownc=1) | [1](http://www.targetscan.org/cgi-bin/vert_61/view_gene.cgi?gs=SHANK3&taxid=9606&members=miR-494&showcnc=1&shownc=1) | 0 | 1 | 0 | 0 | 0 | 0 | 0 |
| [miR-328a/328b-3p](http://www.targetscan.org/cgi-bin/vert_61/view_gene.cgi?gs=SHANK3&taxid=9606&members=miR-328a/328b-3p&showcnc=1&shownc=1) | [1](http://www.targetscan.org/cgi-bin/vert_61/view_gene.cgi?gs=SHANK3&taxid=9606&members=miR-328a/328b-3p&showcnc=1&shownc=1) | 0 | 0 | 1 | 0 | 0 | 0 | 0 |
| [miR-326/330/330-5p](http://www.targetscan.org/cgi-bin/vert_61/view_gene.cgi?gs=SHANK3&taxid=9606&members=miR-326/330/330-5p&showcnc=1&shownc=1) | 0 | 0 | 0 | 0 | [1](http://www.targetscan.org/cgi-bin/vert_61/view_gene.cgi?gs=SHANK3&taxid=9606&members=miR-326/330/330-5p&showcnc=1&shownc=1) | 0 | 1 | 0 |
| [miR-125a-3p/1554](http://www.targetscan.org/cgi-bin/vert_61/view_gene.cgi?gs=SHANK3&taxid=9606&members=miR-125a-3p/1554&showcnc=1&shownc=1) | [1](http://www.targetscan.org/cgi-bin/vert_61/view_gene.cgi?gs=SHANK3&taxid=9606&members=miR-125a-3p/1554&showcnc=1&shownc=1) | 0 | 1 | 0 | 0 | 0 | 0 | 0 |
| [miR-299/299-3p/3563-3p](http://www.targetscan.org/cgi-bin/vert_61/view_gene.cgi?gs=SHANK3&taxid=9606&members=miR-299/299-3p/3563-3p&showcnc=1&shownc=1) | 0 | 0 | 0 | 0 | [1](http://www.targetscan.org/cgi-bin/vert_61/view_gene.cgi?gs=SHANK3&taxid=9606&members=miR-299/299-3p/3563-3p&showcnc=1&shownc=1) | 0 | 0 | 1 |
| [miR-28-5p/708/1407/1653/3139](http://www.targetscan.org/cgi-bin/vert_61/view_gene.cgi?gs=SHANK3&taxid=9606&members=miR-28-5p/708/1407/1653/3139&showcnc=1&shownc=1) | [1](http://www.targetscan.org/cgi-bin/vert_61/view_gene.cgi?gs=SHANK3&taxid=9606&members=miR-28-5p/708/1407/1653/3139&showcnc=1&shownc=1) | 0 | 1 | 0 | 0 | 0 | 0 | 0 |
| [miR-335/335-5p](http://www.targetscan.org/cgi-bin/vert_61/view_gene.cgi?gs=SHANK3&taxid=9606&members=miR-335/335-5p&showcnc=1&shownc=1) | 0 | 0 | 0 | 0 | [1](http://www.targetscan.org/cgi-bin/vert_61/view_gene.cgi?gs=SHANK3&taxid=9606&members=miR-335/335-5p&showcnc=1&shownc=1) | 0 | 1 | 0 |
| [miR-185/882/3473/4306/4644](http://www.targetscan.org/cgi-bin/vert_61/view_gene.cgi?gs=SHANK3&taxid=9606&members=miR-185/882/3473/4306/4644&showcnc=1&shownc=1) | 0 | 0 | 0 | 0 | [1](http://www.targetscan.org/cgi-bin/vert_61/view_gene.cgi?gs=SHANK3&taxid=9606&members=miR-185/882/3473/4306/4644&showcnc=1&shownc=1) | 0 | 1 | 0 |
| [miR-543](http://www.targetscan.org/cgi-bin/vert_61/view_gene.cgi?gs=SHANK3&taxid=9606&members=miR-543&showcnc=1&shownc=1) | [1](http://www.targetscan.org/cgi-bin/vert_61/view_gene.cgi?gs=SHANK3&taxid=9606&members=miR-543&showcnc=1&shownc=1) | 0 | 1 | 0 | 0 | 0 | 0 | 0 |
| [miR-485-5p/1698/1703/1962](http://www.targetscan.org/cgi-bin/vert_61/view_gene.cgi?gs=SHANK3&taxid=9606&members=miR-485-5p/1698/1703/1962&showcnc=1&shownc=1) | 0 | 0 | 0 | 0 | [1](http://www.targetscan.org/cgi-bin/vert_61/view_gene.cgi?gs=SHANK3&taxid=9606&members=miR-485-5p/1698/1703/1962&showcnc=1&shownc=1) | 0 | 0 | 1 |
| [miR-340-5p](http://www.targetscan.org/cgi-bin/vert_61/view_gene.cgi?gs=SHANK3&taxid=9606&members=miR-340-5p&showcnc=1&shownc=1) | [1](http://www.targetscan.org/cgi-bin/vert_61/view_gene.cgi?gs=SHANK3&taxid=9606&members=miR-340-5p&showcnc=1&shownc=1) | 0 | 0 | 1 | 0 | 0 | 0 | 0 |
| [miR-342-3p](http://www.targetscan.org/cgi-bin/vert_61/view_gene.cgi?gs=SHANK3&taxid=9606&members=miR-342-3p&showcnc=1&shownc=1) | 0 | 0 | 0 | 0 | [1](http://www.targetscan.org/cgi-bin/vert_61/view_gene.cgi?gs=SHANK3&taxid=9606&members=miR-342-3p&showcnc=1&shownc=1) | 0 | 0 | 1 |
| [miR-197](http://www.targetscan.org/cgi-bin/vert_61/view_gene.cgi?gs=SHANK3&taxid=9606&members=miR-197&showcnc=1&shownc=1) | 0 | 0 | 0 | 0 | [1](http://www.targetscan.org/cgi-bin/vert_61/view_gene.cgi?gs=SHANK3&taxid=9606&members=miR-197&showcnc=1&shownc=1) | 0 | 0 | 1 |

**Table S2** Altered expression profiles of miR-7, miR-34a, and miR-504 in multiple neuropsychiatric disorders.

| **miRNA** | **Reference (# in main text)** | **Neuropsychiatric disorder** | **Expression Change / Tissue** |
| --- | --- | --- | --- |
| miR-7 | Kim *et al*., 2010 (32) | Schizophrenia | Up / Prefrontal cortex |
| miR-7 | Beveridge *et al*., 2010 (31) | Schizophrenia | Up / Prefrontal cortex |
| miR-7 | Garbett *et al*., 2015 (30) | Major depression | Up / Fibroblast |
| miR-34a | Kim *et al*., 2010 (32) | Schizophrenia | Up / Prefrontal cortex |
| miR-34a | Lai *et al*., 2011 (33) | Schizophrenia | Up / Mononuclear leukocyte |
| miR-34a | Smalheiser *et al*., 2014 (34) | Suicide | Down / Prefrontal cortex |
| miR-34a | Bavamian *et al*., 2015 (29) | Bipolar disorder | Up / Cerebellum |
| miR-504 | Kim *et al*., 2010 (32) | Bipolar disorder | Up / Prefrontal cortex |

**Table S3** Summary of statistical analyses for the experiments.

| **Assay/Measurement** | **Values (mean**$\boldsymbol{\pm}$**SEM, n)** | **Statistical test and P values** | **Figure** |
| --- | --- | --- | --- |
| Luciferase assay for miR-7 binding sites | h*SHANK3* WT, control miR (1$\pm$0.01, 6)  h*SHANK3* WT, miR-7 (0.8$\pm$0.02, 6)  h*SHANK3* Mut, control miR (1$\pm$0.03, 6)  h*SHANK3* Mut, miR-7 (1.1$\pm$0.04, 6)  r*Shank3* WT, control miR (1$\pm$0.05, 6)  r*Shank3* WT, miR-7 (0.73$\pm$0.05, 6) | Unpaired two-tailed Student’s t-test, **P<0.01, ***P<0.001 | Figure 1b |
| Luciferase assay for miR-34a binding sites | h*SHANK3* WT, miR-34a (0.87$\pm$0.02, 6)  h*SHANK3* Mut1, miR-34a (0.67$\pm$0.03, 6)  h*SHANK3* Mut2, miR-34a (0.93$\pm$0.03, 6)  h*SHANK3* Mut3, miR-34a (0.88$\pm$0.03, 6)  h*SHANK3* Mut1/2, miR-34a (1.11$\pm$0.04, 6)  h*SHANK3* Mut1/3, miR-34a (0.71$\pm$0.04, 6)  h*SHANK3* Mut2/3, miR-34a (0.96$\pm$0.04, 6)  h*SHANK3* Mut1/2/3, miR-34a (1.14$\pm$0.03, 6) | Unpaired two-tailed Student’s t-test, *P<0.05, **P<0.01, ***P<0.001 | Figure 1c |
| Luciferase assay for miR-504 binding sites | h*SHANK3* WT, control miR (1$\pm$0.02, 6)  h*SHANK3* WT, miR-504 (0.63$\pm$0.03, 6)  h*SHANK3* Mut1, control miR (1$\pm$0.02, 6)  h*SHANK3* Mut1, miR-504 (0.68$\pm$0.01, 6)  h*SHANK3* Mut2, control miR (1$\pm$0.004, 6)  h*SHANK3* Mut2, miR-504 (1.09$\pm$0.03, 6)  h*SHANK3* Mut1/2, control miR (1$.$03$\pm$0.02, 6)  h*SHANK3* Mut1/2, miR-504 (1.07$\pm$0.05, 6) | Unpaired two-tailed Student’s t-test, ***P<0.001 | Figure 1d |
| Luciferase assay for human/rat 3’UTR and miR-34a | h*SHANK3* WT, control miR (1$\pm$0.02, 9)  h*SHANK3* WT, miR-34a (0.81$\pm$0.02, 9)  r*Shank3* WT, control miR (1$\pm$0.06, 9)  r*Shank3* WT, miR-34a (0.97$\pm$0.09, 9) | Unpaired two-tailed Student’s t-test, ***P<0.001 | Figure 1f |
| Luciferase assay for miR-7, miR-34a and miR-504 synergy | h*SHANK3* WT, control miR (1$\pm$0.003, 6)  h*SHANK3* WT, miR-7 (0.79$\pm$0.005, 6)  h*SHANK3* WT, miR-34a (0.71$\pm$0.01, 6)  h*SHANK3* WT, miR-504 (0.68$\pm$0.006, 6)  h*SHANK3* WT, miR-7+miR-34a (0.66$\pm$0.004, 6)  h*SHANK3* WT, miR7+miR-504 (0.63$\pm$0.004, 6)  h*SHANK3* WT, miR-34a+miR-504 (0.62$\pm$0.006, 6)  h*SHANK3* WT, miR-7+miR-34a+miR-504 (0.06$\pm$0.006, 6) | Unpaired two-tailed Student’s t-test, *P<0.05, ***P<0.001 | Figure 1g |
| *SHANK3* 3’UTR mRNA levels | h*SHANK3* WT, miR-7 (1.06$\pm$0.007, 3)  h*SHANK3* WT, miR-34a (1.03$\pm$0.01, 3)  h*SHANK3* WT, miR-504 (0.98$\pm$0.03, 3)  h*SHANK3* WT, miR-7+miR-34a+miR-504 (0.99$\pm$0.03, 3) | Unpaired two-tailed Student’s t-test | Figure 1h |
| Luciferase assay for *SHANK2* and miR-7 | h*SHANK2* WT, control miR (1$\pm$0.01, 6)  h*SHANK2* WT, miR-7 (1.07$\pm$0.01, 6) | Unpaired two-tailed Student’s t-test | Figure 1i |
| Luciferase assay for Shank3 interacting proteins and miR-7, miR-34a and miR-504 | *ABI2*, miR-7 (0.92$\pm$0.05, 6)  *DBNL*, miR-7 (0.87$\pm$0.01, 6)  *PFN2*, miR-7 (0.36$\pm$0.01, 6)  *SPTBN2*, miR-34a (1.26$\pm$0.27, 6)  *SYNPO*, miR-34a (1.11$\pm$0.04, 6)  *ARPC5*, miR-34a (1.08$\pm$0.03, 6)  *WASF1*, miR-34a (1.03$\pm$0.02, 6)  *MAP1A*, miR-34a (1.01$\pm$0.02, 6)  *ITSN1*, miR-34a (0.91$\pm$0.02, 6)  *BAIAP2*, miR-504 (1.09$\pm$0.05, 6)  *DSTN*, miR-504 (0.86$\pm$0.03, 6)  *MAP1A*, miR-504 (0.76$\pm$0.01, 6)  *SPTBN2*, miR-504 (0.67$\pm$0.02, 6) | Unpaired two-tailed Student’s t-test, *P<0.05, **P<0.01, ***P<0.001 | Figure 2c |
| Luciferase assay for *PFN2* and miR-7 | *PFN2* WT, control miR (1$\pm$0.04, 6)  *PFN2* WT, miR-7 (0.27$\pm$0.01, 6)  *PFN2* Mut, control miR (1$\pm$0.04, 6)  *PFN2* Mut, miR-7 (0.92$\pm$0.05, 6) | Unpaired two-tailed Student’s t-test, ***P<0.001 | Figure 2d |
| Luciferase assay for *SPTBN2* and miR-504 | *SPTBN2* WT, control miR (1$\pm$0.02, 6)  *SPTBN2* WT, miR-504 (0.55$\pm$0.01, 6)  *SPTBN2* Mut, control miR (1$\pm$0.03, 6)  *SPTBN2* Mut, miR-504 (0.91$\pm$0.04, 6) | Unpaired two-tailed Student’s t-test, ***P<0.001 | Figure 2e |
| Luciferase assay for miR-7, miR-34a and miR-504 in cultured neurons | h*SHANK3* WT, control miR (1$\pm$0.18, 5)  h*SHANK3* WT, miR-7 (0.6$\pm$0.08, 5)  h*SHANK3* WT, miR-34a (0.42$\pm$0.08, 5)  h*SHANK3* WT, miR-504 (0.17$\pm$0.03, 5) | Unpaired two-tailed Student’s t-test, *P<0.05, **P<0.01 | Figure 3a |
| Luciferase assay for miR-7, miR-34a and miR-504 in cultured neurons | h*SHANK3* Mut, miR-7 (1.05$\pm$0.12, 5)  h*SHANK3* Mut, miR-34a (0.95$\pm$0.09, 5)  h*SHANK3* Mut, miR-504 (0.96$\pm$0.13, 5) | Unpaired two-tailed Student’s t-test | Figure 3b |
| Dendritic protrusion for miR-7 and miR-504 | Total, control miR (58.77$\pm$1.96, 30)  Total, miR-7 (52.38$\pm$1.89, 26)  Total, miR-504 (45.45$\pm$2.82, 16)  Spine, control miR (56.53$\pm$1.84, 30)  Spine, miR-7 (49.1$\pm$1.75, 26)  Spine, miR-504 (41.97$\pm$2.83, 16)  Filopodia, control miR (2.55$\pm$0.65, 30)  Filopodia, miR-7 (3.02$\pm$0.68, 26)  Filopodia, miR-504 (3.59$\pm$0.83, 16) | One-way ANOVA,  Tukey’s multiple comparison test,  *P<0.05, ***P<0.001 | Figure 3c |
| Shank3 construct western blot for miR-7 and miR-504 | Shank3 with 3’UTR, control miR (1$\pm$0.06, 12)  Shank3 with 3’UTR, miR-7 (0.8$\pm$0.08, 12)  Shank3 with 3’UTR, miR-504 (0.55$\pm$0.08, 12)  Shank3 without 3’UTR, control miR (1$\pm$0.04, 12)  Shank3 without 3’UTR, miR-7 (0.96$\pm$0.1, 12)  Shank3 without 3’UTR, miR-504 (1.07$\pm$0.06, 12) | Unpaired two-tailed Student’s t-test, *P<0.05, ***P<0.001 | Figure 3d |
| Dendritic spine for Shank3 rescue | Control miR (55.29$\pm$2.32, 30)  Shank3 with 3’UTR, control miR (65.92$\pm$1.98, 26)  Shank3 with 3’UTR, miR-7 (49.34$\pm$1.65, 26)  Shank3 with 3’UTR, miR-504 (39.12$\pm$2.21, 20)  Control miR (53.17$\pm$1.54, 28)  Shank3 without 3’UTR, control miR (62.84$\pm$1.24, 25)  Shank3 without 3’UTR, miR-7 (54.32$\pm$1.58, 28)  Shank3 without 3’UTR, miR-504 (49.29$\pm$1.29, 20) | One-way ANOVA,  Tukey’s multiple comparison test,  *P<0.05, ***P<0.001 | Figure 3e |
| Luciferase assay for LNA miR-7, miR-34a and miR-504 in cultured neurons | h*SHANK3* WT, LNA control (1$\pm$0.01, 8)  h*SHANK3* WT, LNA miR-7 (1.15$\pm$0.02, 8)  h*SHANK3* WT, LNA miR-34a (1.13$\pm$0.02, 8)  h*SHANK3* WT, LNA miR-504 (1.18$\pm$0.03, 8) | Unpaired two-tailed Student’s t-test, *P<0.05 | Figure 3f |
| Luciferase assay for LNA miR-7, miR-34a and miR-504 in cultured neurons | h*SHANK3* Mut, LNA miR-7 (1.05$\pm$0.03, 8)  h*SHANK3* Mut, LNA miR-34a (1.03$\pm$0.02, 8)  h*SHANK3* Mut, LNA miR-504 (1.08$\pm$0.08, 8) | Unpaired two-tailed Student’s t-test | Figure 3g |
| Dendritic protrusion for LNA miR-7 and miR-504 | Total, LNA control (59.21$\pm$2.29, 20)  Total, LNA miR-7 (70.61$\pm$4.79, 20)  Total, LNA miR-504 (66.55$\pm$2.71, 18)  Spine, LNA control (52.84$\pm$2.37, 20)  Spine, LNA miR-7 (66.3$\pm$4.9, 20)  Spine, LNA miR-504 (64.35$\pm$2.42, 18)  Filopodia, LNA control (6.37$\pm$1.2, 20)  Filopodia, LNA miR-7 (4.18$\pm$1.16, 20)  Filopodia, LNA miR-504 (2.19$\pm$0.62, 18) | One-way ANOVA,  Tukey’s multiple comparison test,  *P<0.05 | Figure 3h |
| Dendritic spine for Shank3 siRNA rescue | LNA control, si-control (50.87$\pm$2.43, 20)  LNA control, si-Shank3 (38.15$\pm$2.44, 20)  LNA miR-7, si-Shank3 (38.24$\pm$2.09, 20)  LNA miR-504, si-Shank3 (40.34$\pm$3.03, 18) | One-way ANOVA,  Tukey’s multiple comparison test,  *P<0.05, **P<0.01 | Figure 3i |
| Luciferase assay for ciRS-7 and miR-7 | h*SHANK3* WT, ciRS-7-ir, control miR (1$\pm$0.01, 6)  h*SHANK3* WT, ciRS-7-ir, miR-7 (0.58$\pm$0.01, 6)  h*SHANK3* WT, ciRS-7, control miR (1.05$\pm$0.02, 6)  h*SHANK3* WT, ciRS-7, miR-7 (0.83$\pm$0.01, 6)  h*SHANK3* Mut, ciRS-7-ir, control miR (1$\pm$0.05, 6)  h*SHANK3* Mut, ciRS-7-ir, miR-7 (1.08$\pm$0.03, 6)  h*SHANK3* Mut, ciRS-7, control miR (1.04$\pm$0.03, 6)  h*SHANK3* Mut, ciRS-7, miR-7 (1.09$\pm$0.02, 6) | Unpaired two-tailed Student’s t-test | Figure 3j |
| Dendritic protrusion for ciRS-7 | Total, ciRS-7-ir (50.25$\pm$1.95, 20)  Total, ciRS-7 (64.27$\pm$4.39, 15)  Spine, ciRS-7-ir (47.89$\pm$1.95, 20)  Spine, ciRS-7 (59.08$\pm$4.27, 15)  Filopodia, ciRS-7-ir (2.49$\pm$0.58, 20)  Filopodia, ciRS-7 (5.19$\pm$1.34, 15) | Unpaired two-tailed Student’s t-test, *P<0.05, **P<0.01 | Figure 3k |
| Endogenous synaptic proteins for lentiviral expression of miR-504 | Shank3, miR-control (1$\pm$0.04, 3)  miR-504 (0.8$\pm$0.05, 3)  Shank2, miR-control (1$\pm$0.05, 3)  miR-504 (1.09$\pm$0.06, 3)  PSD-95, miR-control (1$\pm$0.04, 3)  miR-504 (0.94$\pm$0.05, 3) | Unpaired two-tailed Student’s t-test, *P<0.05 | Figure 4b |
| Expression of Shank3 constructs in cultured neurons | Shank3 with 3’UTR, control miR (1.00$\pm$0.05, 15)  Shank3 with 3’UTR, miR-7 (0.82$\pm$0.02, 15)  Shank3 with 3’UTR, miR-504 (0.76$\pm0.04$, 15)  Shank3 without 3’UTR, control miR (1.00$\pm$0.09, 14)  Shank3 without 3’UTR, miR-7 (0.98$\pm$0.04, 14)  Shank3 without 3’UTR, miR-504 (0.90$\pm$0.03, 15) | One-way ANOVA,  Tukey’s multiple comparison test,  **P<0.01, ***P<0.001 | Figure S2 |

**Figure S1** The secondary structures of *SHANK3* (a) and *SHANK2* (b) 3’UTRs predicted by *RNAfold* (<http://rna.tbi.univie.ac.at/cgi-bin/RNAfold.cgi>) according to the minimum free energy. The color code represents base-pair probabilities.


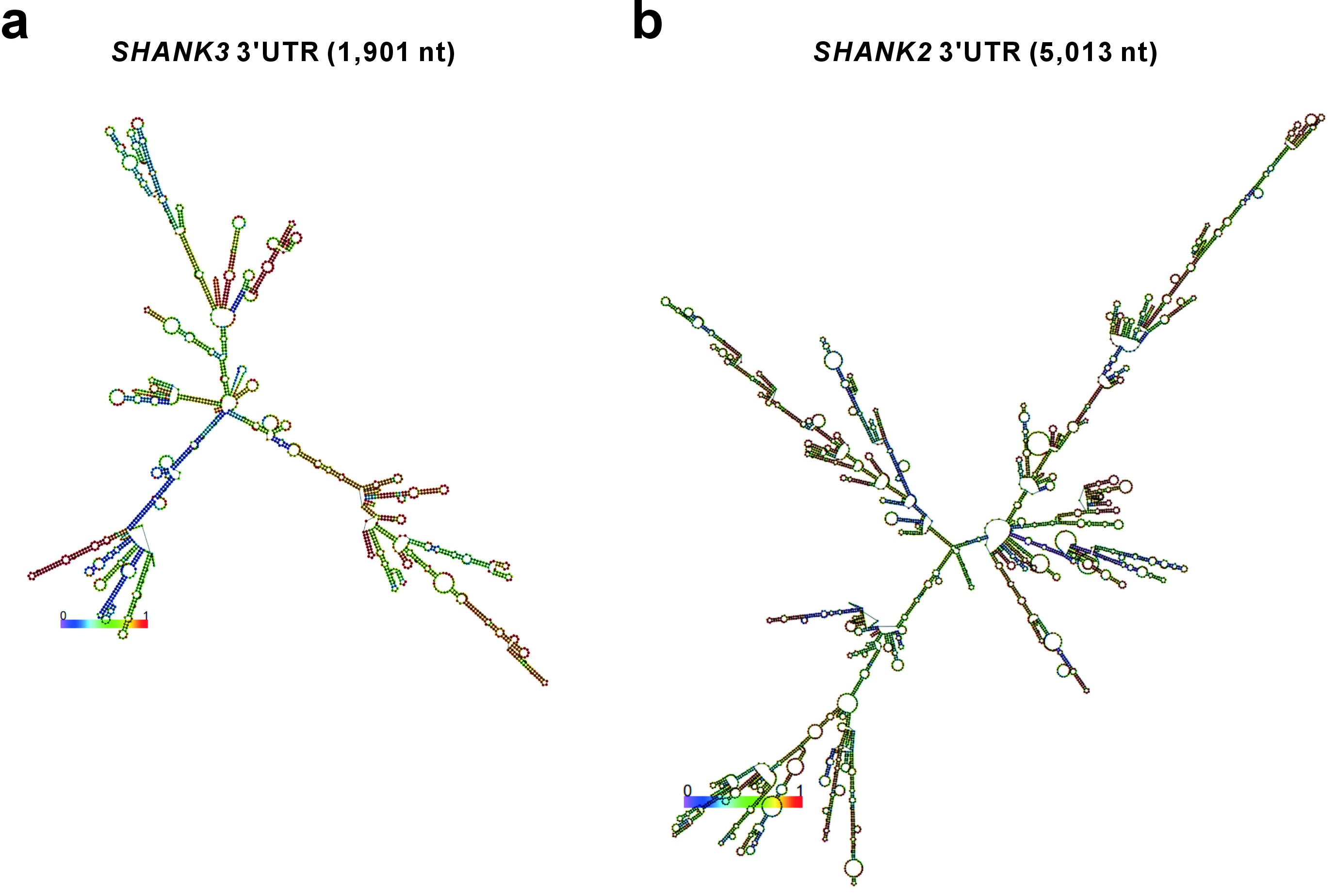


**Figure S2** The expression of Shank3 construct with 3’UTR, but not that without 3’UTR, was significantly decreased by miR-7 and miR-504 in mouse cultured neurons. Statistical analyses are in Table S3.

**
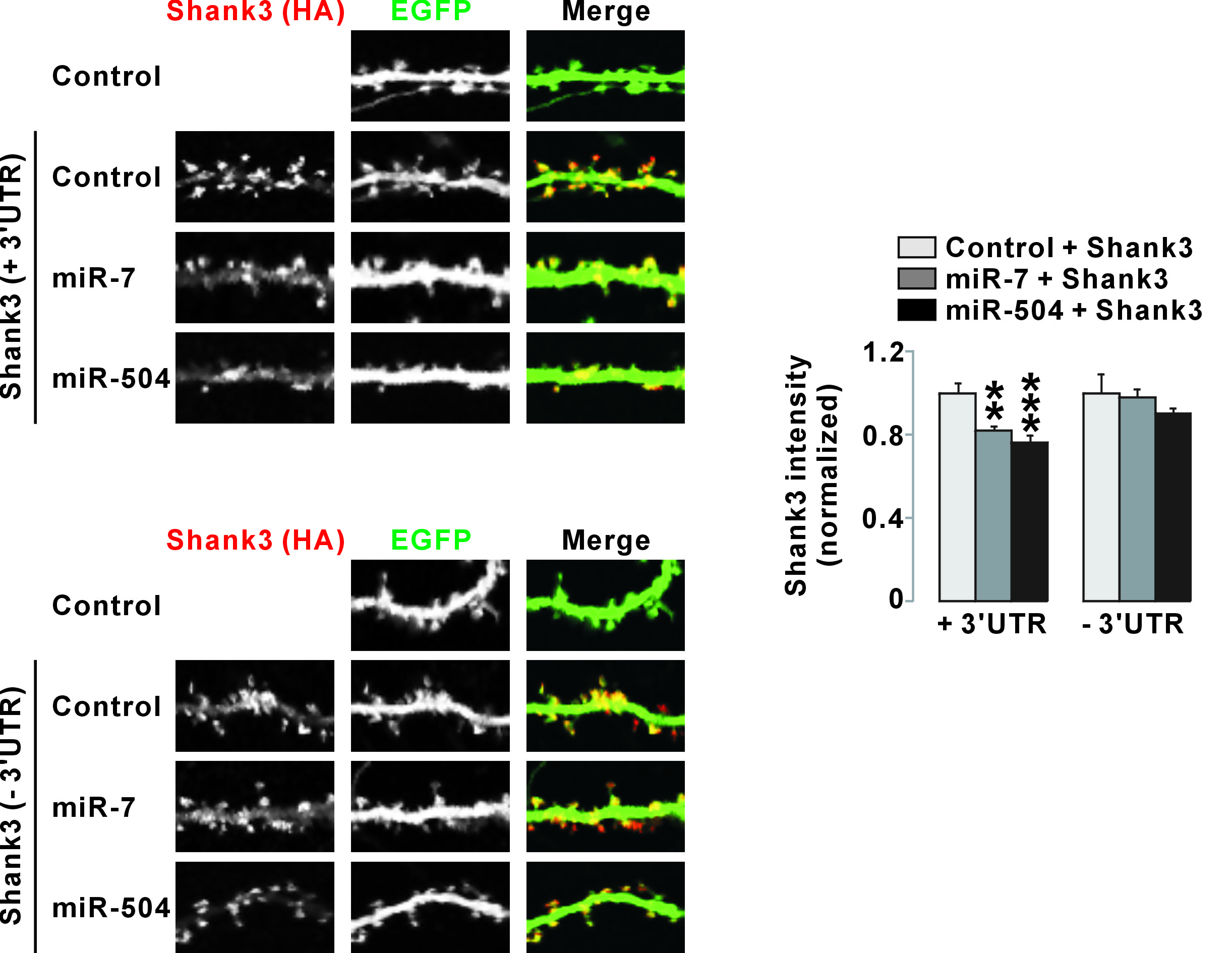
**

**Figure S3** The ten major Shank3 isoforms (Wang *et al*. Molecular Autism 2014, 5:30) (a), and the nucleotide sequence of the last exon (exon 22) of *Shank3* gene (b). The Shank3 isoforms containing SAM domain at the C-terminus are labeled with red color.


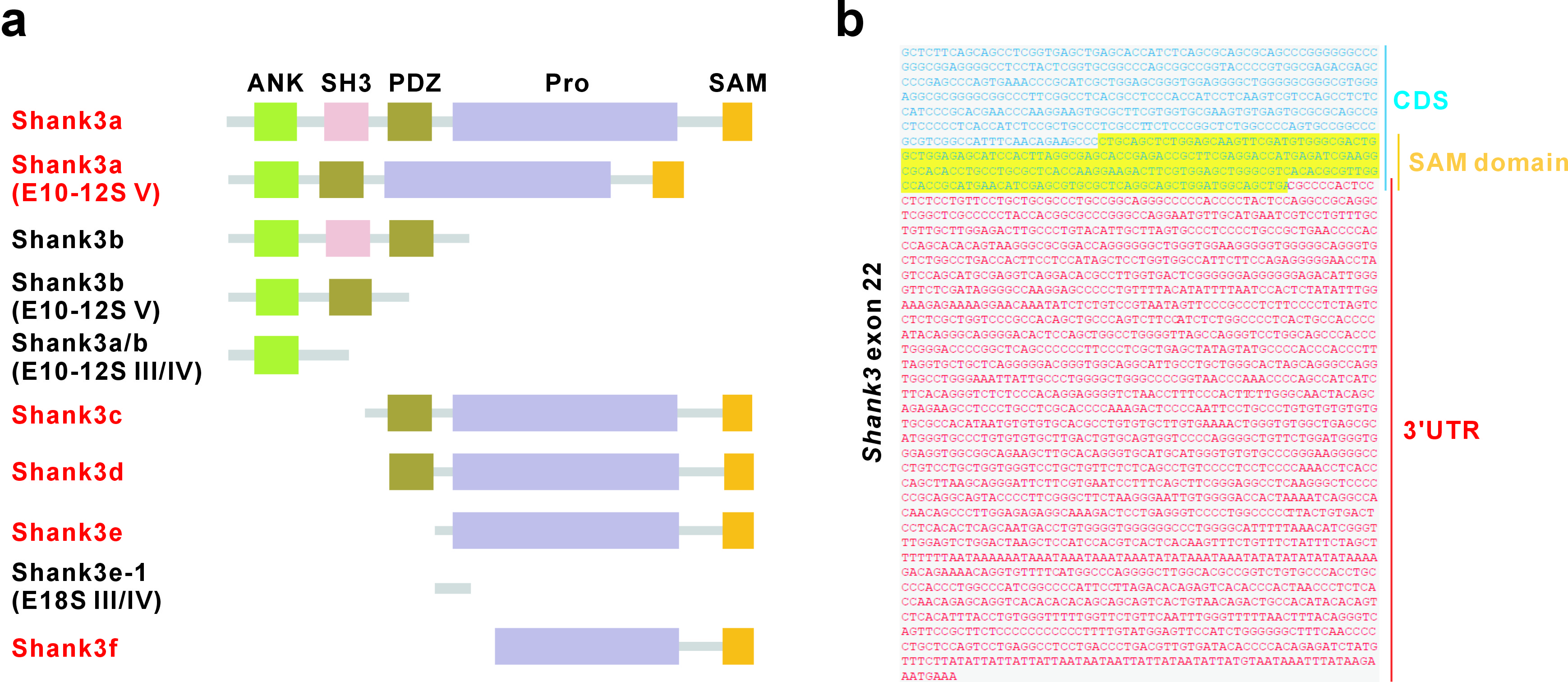

Supplement: Additional file 1: Table S1. — The putative miRNA families targeting the SHANK3 3′UTR predicted by TargetScan (release 6.2). The number of binding sites for each miRNA family, and the binding type (8mer, 7mer-m8, or 7mer-1A) are described. Table S2. Altered expression profiles of miR-7, miR-34a, and miR-504 in multiple neuropsychiatric disorders. Table S3. Summary of statistical analyses for the experiments. Figure S1. The secondary structures of SHANK3 (a) and SHANK2 (b) 3′UTRs predicted by RNAfold (http://rna.tbi.univie.ac.at/cgi-bin/RNAfold.cgi) according to the minimum free energy. The color code represents base-pair probabilities. Figure S2. The expression of Shank3 construct with 3′UTR, but not that without 3′UTR, was significantly decreased by miR-7 and miR-504 in mouse cultured neurons. Statistical analyses are in Additional file 1: Table S3. Figure S3. The ten major Shank3 isoforms (Wang et al. Molecular Autism 2014, 5:30) (a), and the nucleotide sequence of the last exon (exon 22) of Shank3 gene (b). The Shank3 isoforms containing SAM domain at the C-terminus are labeled with red color. (DOCX 3382 kb) [file 13041_2015_165_MOESM1_ESM.docx]
